# Perceptions of patients with chronic obstructive pulmonary disease and their health care providers towards using mHealth for self-management of exacerbations: a qualitative study

**DOI:** 10.1186/s12913-018-3545-4

**Published:** 2018-10-04

**Authors:** Y. J. G. Korpershoek, S. C. J. M. Vervoort, J. C. A. Trappenburg, M. J. Schuurmans

**Affiliations:** 1 Research Group Chronic Illnesses, University of Applied Sciences Utrecht, Heidelberglaan 7, 3584 CS, Utrecht, PO Box 12011-3501, AA The Netherlands; 2 Julius Center for Health Sciences and Primary Care, University Medical Center Utrecht, Utrecht, The Netherlands; 3 Cancer Center, University Medical Center Utrecht, Utrecht, The Netherlands; 4 Education Center, UMC Utrecht Academy, University Medical Center Utrecht, Utrecht, The Netherlands

**Keywords:** COPD, Exacerbation, Qualitative research, Thematic analysis, Focus groups, Self-management, Self-care

## Abstract

**Background:**

Self-management of exacerbations in COPD patients is important to reduce exacerbation impact. There is a need for more comprehensive and individualized interventions to improve exacerbation-related self-management behavior. The use of mobile health (mHealth) could help to achieve a wide variety of behavioral goals. Understanding of patients and health care providers perspectives towards using mHealth in promoting self-management will greatly enhance the development of solutions with optimal usability and feasibility. Therefore, the aim of this study was to explore perceptions of COPD patients and their health care providers towards using mHealth for self-management of exacerbations.

**Methods:**

A qualitative study using focus group interviews with COPD patients (*n* = 13) and health care providers (HCPs) (*n* = 6) was performed to explore perceptions towards using mHealth to support exacerbation-related self-management. Data were analyzed by a thematic analysis.

**Results:**

COPD patients and HCPs perceived mostly similar benefits and barriers of using mHealth for exacerbation-related self-management. These perceived benefits and barriers seem to be important drivers in the willingness to use mHealth. Both patients and HCPs strengthen the need for a multi-component and tailored mHealth intervention that improves patients’ exacerbation-related self-management by determining their health status and providing adequate information, decision support and feedback on self-management behavior. Most importantly, patients and HCPs considered an mHealth intervention as support to improve self-management and emphasized that it should never replace patients’ own feelings nor undermine their own decisions. In addition, the intervention should be complementary to regular contact with HCPs, as personal contact with a HCP was considered to be very important. To optimize engagement with mHealth, patients should have a positive attitude toward using mHealth and an mHealth intervention should be attractive, rewarding and safe.

**Conclusions:**

This study provided insight into perceptions of COPD patients and their HCPs towards using mHealth for self-management of exacerbations. This study points out that future mHealth interventions should focus on developing self-management skills over time by providing adequate information, decision support and feedback on self-management behavior and that mHealth should complement regular care. To optimize engagement, mHealth interventions should be attractive, rewarding, safe and tailored to the patient needs.

**Electronic supplementary material:**

The online version of this article (10.1186/s12913-018-3545-4) contains supplementary material, which is available to authorized users.

## Background

Chronic Obstructive Pulmonary Disease (COPD) is a highly prevalent chronic disease worldwide and is associated with a significant burden on both patients and society [1, 2]. The natural course of COPD is interrupted by exacerbations, defined as ‘a sustained worsening of patients’ respiratory symptoms, which are beyond normal day-to-day variability and may warrant medical treatment’ [3]. These exacerbations are associated with decline in lung function and quality of life [4–6], increased mortality [7], and increased healthcare use [8].

The absence of an adequate imminent exacerbation marker requires to focus on supporting COPD patients in developing self-management skills to be able to adequately detect exacerbations and to take prompt actions and thereby reduce the impact of exacerbations [9, 10]. A recent Cochrane review shows that self-management interventions including exacerbation action plans, are associated with improvements in quality of life and lower probability of hospital admissions [11]. By focusing on exacerbations more specifically, another recent Cochrane review shows that solely exacerbation action plans also reduce in-hospital health care utilization and increase exacerbation treatment [12]. However, a more detailed view on these aggregated results shows that still a substantial proportion of COPD patients barely benefits from these kind of interventions. This might be explained by the ‘one size fits all’ and static approach regarding design, intensity and mode of delivery, the sole focus on exacerbation detection and taking action and suboptimal use of interventions [12–14].

To further reduce the impact of exacerbations, a more comprehensive, dynamic and individualized strategy is needed that improves the full spectrum of exacerbation-related self-management behavior [11, 14, 15]. Since previous studies have shown that self-management interventions only improve patient outcomes when changing self-management behavior of COPD patients [16, 17], it is important that COPD self-management interventions aim at motivating, engaging and supporting patients to positively adapt their behaviors [15]. To address this complex challenge, the use of mobile health (mHealth) might be a solution [18, 19]. In the past decade, research has increasingly focused on using mobile technology for self-management purposes in chronic lung diseases [20, 21]. Through mHealth, several accessible and essential real-time elements can be added to current static self-management support enlarging the intensity and set of options in communication, monitoring and delivery of therapeutic solutions and allowing delivery of self-management support when and where needed. Furthermore, mHealth creates opportunities to include effective behavior change techniques to enhance patient motivation for self-management, for example by monitoring the behavior and providing feedback and rewards [18]. By using mHealth, self-management support could be more individualized and more dynamic and intensive therapeutic stimuli can be provided that fit current health status. To allow development of effective mHealth interventions and optimize engagement with these interventions, the patient’s needs and current healthcare context should be thoroughly investigated during the intervention development stage [18, 22, 23]. Furthermore, a deep understanding of both patients and health care providers perspectives towards using mHealth for exacerbation-related self-management will greatly enhance developing solutions with optimal usability and feasibility [24]. Although mHealth technology is of growing interest in health care, patient and health care provider perspectives towards using mHealth for self-management are relatively unexplored. Little is known about COPD patients and health care providers willingness to use mHealth for self-management of exacerbations, their perceptions towards potential benefits and barriers of using mHealth and their preferences regarding potential content of mHealth interventions.

The main aim of the study was to explore perceptions of COPD patients and their health care providers towards using mHealth for self-management of exacerbations. More specific, the objectives were to:

- Explore both COPD patients and health care providers willingness to use mHealth for self-management of exacerbations.
- Identify potential benefits and barriers of using mHealth for self-management of exacerbations.
- Explore needs and preferences of both COPD patients and health care providers regarding content of an mHealth intervention for self-management of exacerbations.

## Methods

### Study design

A qualitative study using focus group interviews with COPD patients and health care providers was performed to elicit ideas, thoughts and perceptions towards using mHealth to support exacerbation-related self-management [25]. Focus groups interviews were considered to be relevant since interaction between participants may lead to mutual understandings and ideas towards using mHealth [26]. This study was approved by the Medical Ethics Research Committee of the University Medical Centre Utrecht (15–134/C).

### Study population

A purposive sample of Dutch patients with a clinical diagnosis of COPD, who had experienced at least one exacerbation in the last twelve months, was selected from two general practices, two physiotherapy practices and one hospital in the region of Utrecht. Patients had to meet the inclusion- and exclusion criteria as described in Table 1. Maximum variation sampling was used to create a large diversity in patients’ age, sex, COPD severity and mobile device skills, aiming to increase the likelihood of reflecting different perspectives in the findings. Furthermore, a purposive sample of health care providers (HCPs) was selected according to the inclusion- and exclusion criteria as described in Table 1. Diversity in professional disciplines, years of experience in COPD care and age was pursued using maximum variation sampling.

**Table 1:** Inclusion and exclusion criteria

Participants	
Patients with a clinical diagnosis of COPD	
*Inclusion*	*Exclusion*
• Age > 40 years	• Severe problems with vision or hearing
• FEV1/FVC ratio < 70%	• Diagnosed with cognitive impairments
• GOLD stage ≥2, Spirometry FEV1 < 80% predicted	• Primary diagnosis of asthma, cardiac disease or other major functionally limiting diseases
• ≥ 1 exacerbation^a^ in the last 12 months prior to entering this study (to ensure adequate recall of their experience of an exacerbation).	• Life expectancy ≤ three months
Health care providers	
*Inclusion*	*Exclusion*
• Having a patient-health care provider relationship with COPD patients	NA
• Supporting COPD patients in self-management.	
• At least 1 year experience in COPD care.	

Abbreviation: *NA* = not applicable; *GOLD*, Global Initiative for Chronic Obstructive Lung Disease; *FEV*_1_, forced expiratory volume in 1 s; *FVC*, forced vital capacity

^a^An exacerbation was defined as a period of symptom deterioration in which use of a course of corticosteroids and/or antibiotics was required or hospitalization was necessary; Clinical diagnosis was based on data from Global Initiative for Chronic Obstructive Pulmonary Disease [1].

### Recruitment and informed consent

Patients were informed about the study by their HCP and received written study information. A patient willing to participate, was contacted by the researcher to provide further information and to verify willingness to participate in the study. Two patients were approached for this study during their participation in a previous study focusing on perceptions towards exacerbation-related self-management [27], and one patient was informed about the study by the Dutch patient society for lung disease. HCPs were informed about the study by the researcher (YK) by email and received written study information attached. Their willingness to participate in the study was coordinated by email. Written informed consent was obtained from all participants. Three focus groups were conducted between June 2015 and May 2016.

### Data collection

Focus group interviews (*n* = 6–8) were performed using a topic list for formulating open questions [26]. After a general introduction of the focus group aim, procedure and explanation of important terminology used in the focus group, the following broad topics were discussed in all focus groups investigating both patient and HCP perspectives: Experience with mobile devices (patients) or mHealth interventions (HCPs), patient needs towards exacerbation-related self-management support, perceptions towards use of mHealth for self-management of exacerbations (potential benefits and barriers), preferences regarding content of an mHealth intervention. After asking participants about their preferences regarding potential content in an open manner, the following predetermined topics were discussed: self-monitoring of symptoms, access to data by a HCP and contact with a HCP, feedback on behavior, education, decision making and reminders. In the focus group with patients specific emphasis was placed on their needs regarding self-management support. Contrastingly, the focus group with HCPs focused specifically on perspectives towards their own role in supporting patients’ self-management using mHealth. Both topic lists are detailed in an additional file (see Additional file 1).

All focus group interviews were conducted by an experienced moderator not part of our research team. In total, two independent moderators were involved in this study. Their role was to introduce the topics, encourage participants to share their thoughts, ask additional questions to clarify participants’ expressions. Furthermore, assistant moderators were present at each focus group to coordinate practical issues and one of more secretaries were present to describe group interactions, reflect on methodological issues and capture initial ideas on themes in memos.

The focus groups were held at the University Medical Center of Utrecht and each focus group lasted approximately two hours. All interviews were audiotaped. Baseline characteristics of participants were gathered after the focus group interview by a short questionnaire. All data were encoded to guarantee anonymity. To increase methodological quality of the study, an expert on qualitative research with a nursing background was involved in the process of data collection and data analysis (SV).

### Data-analysis

Data were analysed according to a thematic analysis as described by Braun&Clarke, to identify, analyse and report themes within the data [28]. Data were analysed by two independent researchers (YK&SV). After the focus group interviews, initial ideas were captured in memos by the researcher (YK). All interviews were transcribed verbatim. Data analysis was supported by NVivo 10.0 software (QSR International Pty Ltd. Version 10, 2012).

Firstly, the two researchers read the transcripts in its entirety to get an overall picture and summarized information obtained with regard to the research objectives. Secondly, the interviews were reread in more detail, initial codes were connected to meaningful paragraphs by both researchers and discussed afterwards to reach consensus. Third, identified codes were brought under potential themes and, subsequently, reviewed for correspondence to the coded paragraphs. Finally, potential themes were further defined and clear definitions were generated [28]. The process of analysis was supported by memo-writing.

Credibility of the study was enhanced by emphasizing the aim to learn from participants and an open, non-judgmental attitude of the moderator during the focus group. Furthermore, discussions with two experts on the interpretation of data (JT&MS) contributed to the study’s credibility [29]. Transcription of the focus group interviews and researcher triangulation in all phases of the study diminished chances to bias.

## Results

A total of *n*=13 COPD patients (4 male and 9 female) and *n*=6 HCPs (4 male and 2 female) participated in the study. Baseline characteristics of the participants are presented in Tables 2 and 3. Maximum variation in COPD patients was reached in disease severity, age, sex and mobile technology use. Self-reported exacerbations ranged from less than one until 5 self-reported exacerbations per year (Table 2). The focus group with health care providers consisted of nurses, a general practitioner, a resident in pulmonology and a respiratory physiotherapist. Their age varied widely as well as their experience in COPD care.

**Table 2:** Baseline characteristics of patients

ID	Age range	Living situation	Education level^a^	Smoking	GOLD stage^b^	Exacerbations/ year^c^	Mobile technology use
P01	50–59	With life partner	High	Former	2/3^d^	3	Smartphone & tablet
P02	50–59	With life partner	Low	Former	2	5	Smartphone & tablet
P03	50–59	–	–	–	2/3	–	Smartphone & tablet
P04	60–69	With life partner	Low	Former	4	3	Tablet
P05	60–69	Alone	High	Current	4	< 1	Smartphone & tablet
P06	60–69	With life partner	High	Former	3	> 3	None
P07	70–79	Alone	High	Former	2	2	Tablet
P08	80–89	With life partner	Medium	Former	2	3	None
P09	50–59	With life partner	Medium	Former	4	3	Smartphone & tablet
P10	60–69	With children	High	Former	3	1	Smartphone & tablet
P11	60–69	With life partner	High	Former	4	> 3	Tablet
P12	40–49	With life partner	Medium	Former	3	3	Tablet
P13	70–79	With life partner	Low	Former	2	–	None

Abbreviation: *GOLD*, Global Initiative for Chronic Obstructive Lung Disease

^a^Low = primary school through vocational training, medium = secondary school or vocational training, high = college or university degree

^b^according to GOLD classification in medical chart;

^c^amount of exacerbations determined by amount of prescriptions of corticosteriods and/or antibiotics for worsening of lung symptoms, estimated by patients themselves;

^d^patient self-reported GOLD stage; **−** = missing data

**Table 3:** Baseline characteristics of health care providers

ID	Age range	Profession	Setting	Work experience in year ranges	Patient GOLD category most frequently cared for^c^	mHealth experience in daily pratice
H01	20–29	Respiratory nurse	Hospital	0–5	2–3 / 3–4	None
H02	20–29	Nurse at lung department	Hospital	0–5	3–4	None
H03	40–49	Respiratory nurse specialist	Hospital	16–20	3–4	Yes
H04	30–39	Resident in pulmonology	Hospital	6–10	3–4	None
H05	30–39	Respiratory physiotherapist	Physiotherapy practice	11–15	2–3 / 3–4	None
H06	50–59	General practitioner	General practice	21–25	1–2 / 2–3	None

Abbreviation: *GOLD*, Global Initiative for Chronic Obstructive Lung Disease

^c^classified by GOLD stage 1–2, 2–3 and/or 3–4.

The results of the focus group interviews are described in the following paragraphs and further illustrated by quotes of the participants in Tables 4 and 5 (Q references in the text refer to quotes of specific themes in Tables 4 and 5).

**Table 4:** Illustrative quotes related to patient needs and benefits and barriers of using mHealth for self-management

Theme	Quote	
Patient needs toward self-management support		
*Being heard by a HCP*	*Q1*	P02: “The doctor said ‘There’s nothing I can do for you because you have the flue’ […} as result that I became really ill. […] Yes, I always get really emotional when I’m not being heard. It’s really important that you are being heard.” H06: “Because for the patient, I believe being heard is what’s needed the most.”
*Adequate support from a HCP*	*Q2*	P04: “It was so bad that I called the pulmonary nurse at the hospital on Friday. She said ‘Yes, Madam, the flue is going around, so just take some extra inhalations’ and that’s how I entered my weekend. I can’t believe I agreed with it. Then in the afternoon I called my own pulmonary nurse, she’s always really helpful. She immediately sent the prescription for antibiotics to the pharmacy, but it was actually too late and I ended up in hospital.” H05: “Patients really appreciate a little extra support. If you tell them that it’s all right to call and ask for help, they will do that much more easily.”
*Tools to substantiate health status*	*Q3*	P11: “When it’s really bad you can feel it. But most of the time it just grows consistently. It would be really nice if earlier on I would feel it, so I can tell myself to be careful. Then it would be possible to adjust my behavior, my energy and based on those two, my medication.”
*Information on actions*	*Q4*	P01: “At the time, I thought it wasn’t too bad. Well it actually is bad nowadays. Back then I should have known what to do. […] I wish I would have had the information at that time about how I could recognize that I am not doing well […] how I can manage a threating attack.”
*Social support*	*Q5*	P12: “I am always late as I just explained. So it’s usually my husband who tells me ‘If you don’t go now, I will take care of it’. You know, like, then I will make the call.”
*Elimination of barriers in health care system*	*Q6*	P09: “The discussion I have with a doctors assistant like: ‘You need to come in’, and I tell them that I’m too short of breath and they tell me: ‘We understand, but we can’t just give you medication, so you have to come in and see the doctor.’ That’s the point that I give up and tell them to ‘never mind’ and I just hang up the phone. […] Well it’s just not possible to directly see a pulmonologist, is it?”
Benefits of using mHealth for self-management		
*Contributes to awareness of symptom deterioration*	*Q7*	P07: “I would be willing to fill out a questionnaire but only so that I can get more insight into what it actually is that I have. […] It would be a nice tool to help you point out what you feel. It would be like ‘I feel like this’ but also ‘this could be the source of your problem’ so what you should do is take some extra puffs, something like that.” H02: “Early detection of an exacerbation, that the app could help the patients find out if that is what is happing. That it would be possible for the patients to insert information on symptoms into the app every time they experience upcoming symptoms. So that the app can be supportive in detecting an exacerbation.”
*Demonstrate and underpin current health status*	*Q8*	P10: “It would help me in convincing the general practitioner, because then I could support what I feel. Like, ‘I feel this, and the app indicates this as well’” P01: “So that in that moment I can say, ‘Listen, I’ve kept track of my symptoms and I have COPD. Look, the app is giving me a warning’. Maybe that would be supportive in being heard.”
*Supports taking adequate actions*	*Q9*	P08: “When I would be really short of breath and I would feel really bad, what I would have to do.” H05: “Maybe it would help them in deciding that this is the moment to take action and stimulate them to do so. The application could reduce the threshold to take that step.”
*Supports prompt health care contact*	*Q10*	H02: “An app could give an extra sign to patients when they’ve reached a point that their symptoms are so bad, they have to call. That could support patients at times when they feel guilty for calling or asking for help, because the app said that it was all right to make a call.”
Barriers of using mHealth for self-management		
*Avoiding confrontation with the disease*	*Q11*	P01: “I don’t want to be too much confronted with being ill. I’m still working and I don’t know, I just want to be able to do that for as long as possible. So I don’t want to be thinking about being ill all the time.” P07: “On one hand you really want to know and on the other hand you really don’t!” H01: “They don’t want it to rule their day. If it’s going well, it’s going well.”
*Preference for personal contact*	*Q12*	P12: “Yes, I’m leaning towards 1 on 1 contact, I mean personal contact. That’s the most important for me.” P05: “Being heard is necessary while being at the doctor, that’s what I think… An app doesn’t support that.”
*Difficulties with displaying feelings in application*	*Q13*	P09: “I would fill out a questionnaire but it’s a bit black and white in my opinion […] How do I feel? Well, I feel ‘so so’. How do you explain ‘so so’?” H06: “Yes, how sensitive is it when you ask ‘How do you feel today?’ H05 responds:” Indeed, that’s the question, how well able is a patient to give it a rating? H06: “Yes, in that case you need to have kind of a list, if it has to be valid…”
*Lack of trust in advice through mHealth*	*Q14*	P10: “The dangerous thing is that the app can report something differently than how I’m feeling. In that case, the app prevents me from taking actions I would now do, that’s the downside. I really have the idea that you are the most capable yourself of feeling how you are doing at a specific point.” P05: “Well…It would surprise me if an app is capable of advising me what to do […] How could an app think for me about what is the right thing to do at a certain point in time? And whether or not I should take pills or get a course of medicine…?”
*Having adequate self-management skills*	*Q15*	P01: “Well to be honest, I have the feeling that with the knowledge and support I have now, I’m capable to act in case of a upcoming exacerbation…So I don’t have the feeling that it would help me a lot.”
*Lack of enthusiasm for mHealth by HCPs*	*Q16*	P02: “Because I have the idea that pulmonologists, and everybody, are not waiting for it. Their enthusiasm for these things is a rare thing. Let me put it this way, they’re having their hands full already.”
*Not all patients are eligible*	*Q17*	H06: “My great concern would be about who is going to use the app.” H03: “I think that patients with frequent exacerbations just won’t use or even install the app…[…] I had a patient who could not be motivated or be stimulated at all. So no… that’s really hard.”
*Could delay health care contact*	*Q18*	H06: “A patient should not spent time on reading a forum to find out what other patients with COPD would do.”

**Table 5:** Illustrative quotes related to preferences regarding an mHealth intervention and facilitators for engagement with mHealth

Theme	Quote	
Preferences regarding content of an mHealth intervention		
*Providing information*	*Q19*	P05: “Well to be specific, I think that the information on symptoms would be a really good one…Whether your symptoms are severe or not, or whether it’s in an early stage or not. I really think that’s what’s really important.”
*Action plan for decision support*	*Q20*	P11: “Sometimes when you are so short of breath, you forget things because of that, or you skip a step…and eh…For me, it would be useful to have the right steps clear for myself or to be able to adjust these steps for myself, right? That you follow the right steps in case you are short of breath...What is it? What to do? How to breath? Is there anyone you should consult? Who to consult? And what kind of medicine?” H02: “So when the app gives a certain score or something like that, or you get a specific score over two days, that the app gives you a fitted advice based on that, like: ‘You need to contact your general practitioner or pulmonary nurse’.”
*Reminders*	*Q21*	P03: “For people who are recently diagnosed with COPD, the reminders could be useful. They won’t have to re-invent things for themselves, like I had to do.” H02: “Yes, for example, I think of reminders…Maybe for recently diagnosed patients, for example to use their inhaler or medication.”
*Self-monitoring of symptoms*	*Q22*	P01: “To be honest, I don’t know if I would use it…[...] With all the respect, we are talking about some kind of app. I just have my doubts because I actually want to be confronted with my illness as less as possible…” P05: “Well, it depends…on the length of the questionnaire you need to fill out, that’s what I think.” H06: “I think that after a while, a lot of patients just say ‘it’s going fine or everything is okay’. And why would you fill out the questionnaire then?”
*Information exchange with HCP*	*Q23*	P10: “What I don’t want is that the app communicates directly with my caregiver. I want to be able to control that myself. That’s a decision I want to make. So when I think it’s important, I believe I should be able to communicate that. But I want to be the one that can make that decision to do so.”
Facilitators for engagement with mHealth		
*Targeting and tailoring of mHealth*	*Q24*	P01: “I wonder if you shouldn’t make a distinction in the app between patients with mild COPD and patients with more severe COPD.” H01: “If so, it needs to be personalized.” H06: “On the other side, it needs to be manageable as well [...] For every healthcare professional. Maybe I do know of my own patients how the app has been tailored, but what if my patient comes to see you? You should be able to directly see what it is about as well.”
*Attractiveness*	*Q25*	H05: “I think it’s important to focus on the essence of the application. In my opinion, it needs to be small and simple with a very specific goal.” H03: “It needs to be manageable for the patient category. When you think about the elderly, it could already be difficult with mobile devices. It shouldn’t be too difficult with all kinds of dots and lines. Then I think that they will stop using the app soon.”
*Positive confirmation or rewards*	*Q26*	P01: “What might stimulate me to use the app, is to insert positive things, like: ‘I am seeing a physiotherapist twice a week, well done!’” H04: “A reward is the best thing that works of course. […] A reward structure, so that when you have filled in things correctly, you will receive a compliment. […] People are simple, just something with illustrations: positive confirmation.”
*Focus on patients own decisions*	*Q27*	P09: “But it’s possible to use the app for advice right? You always remain in control right?” (Moderator asks: ‘Would you like to receive an advice based on the questions you answered?’) P10: “Well maybe if it’s really an advice [...] I don’t want to have to; I want to be able to make my own decision to do it yes or no.”
*Having skills and a positive attitude*	*Q28*	H06: “You need to be open minded.” H03: “I noticed in my own practice (while testing another app) that half, or maybe more than half, of the patients couldn’t deal with it and were also not willing to use it. The other patients, a smaller group, are really enthusiastic about it and highly motivated. In that case, it doesn’t matter that much what kind of self-management intervention you offer from a distance.” (Moderator asks: “And what was the reason for that difference?”) “I think it depends on their cognitive skills, how they cope with their disease.”
HCP perspectives towards their role regarding mHealth use		
*Adequate positioning of responsibilities*	*Q29*	H03: “You try to leave it with the patient, but then you notice that it just doesn’t always work that way. And that you yourself need to take a proactive role to reach out to the patient again” H04: “It’s preferable to give patients more responsibility in their self-management and that you try to work towards that, so that should be the aim.[…] That includes making proper arrangements about how it’s going to be when it works out, and of course emphasize that when things don’t work out, they can always count on help.”
*Perceived control by increasing patient responsibilities*	*Q30*	H04: “That’s rewarding for patients. When you report your symptoms in the app and it leads to advice and you reach out for help. Then something happens which makes that you can prevent things. As a health care provider you can say at the beginning ‘Well you’re in control. If you use the app, you can experience the benefits yourself.’ And with that the app becomes more important.”
*Monitoring by an HCP can be unsafe*	*Q31*	H06: “I would think that would be dangerous too. Because, well…I read my messages every fifteen minutes. If I don’t do that for a couple of hours then I could miss things, you don’t want that to happen [...]. For instance, you insert a really bad value, like you have a fever or your saturation is low… when patients don’t get a call from the nurse or doctor at that time, they could think ‘Well it’s probably not that important.’”
*Time investment for HCP*	*Q32*	H02: “I really wonder if as a nurse or primary care nurse you would really have the time for that. Because when I think about self-management, it’s the patient’s responsibility to do something with the information he or she gets out of the app, instead of the nurse or somebody else receiving notifications and having to call all the patients.”
*Goal of self-management*	*Q33*	H01: “I really wonder if it’s still self-management then…” H05: “Especially the danger of patients thinking they can have a passive role and don’t have to do anything themselves anymore. That, of course, does not correspond with the goal of self-management.”

### Experience with mobile technology

Mobile technology use was introduced as either smartphone or tablet use. Some patients not using these mobile devices did use a computer or laptop and were encouraged to think about opportunities to support self-management through these devices. Most common purposes of mobile technology use by patients were: phoning, social media, seeking information on the internet, application use, messaging and gaming. Mobile technology was barely used for health purposes. Two patients had some experience with health applications by participating in previous eHealth research. According to patients, most common advantages of mobile technology use were the low threshold to use mobile technology and opportunities for social networking. Experienced disadvantages were: privacy sensitiveness and limited usability since mobile devices are too small to navigate properly. Although most patients were currently using mobile devices, some patients did not have a mobile device. Reasons not to use mobile devices were: no interest in mobile technology, poor digital skills and high costs. By asking HCP’s about their experience with mobile technology in health care, only one HCP mentioned to have experience with health applications in daily practice.

### Patient needs toward exacerbation-related self-management support

Before focusing on patient and HCP perspectives towards using mobile technology for exacerbation-related self-management, they were both asked about patient needs toward self-management support. Based on both patient and HCP responses, patient needs varied largely per individual. Although some patients mentioned that they had adequate skills for exacerbation-related self-management, a substantial part mentioned having difficulties with exacerbation detection and taking prompt action. One of the most important needs, according to both patients and HCPs, was that patients are properly heard by their HCP and receive adequate support from their HCP (Q1&2). Having a good relationship with HCPs was considered to be important for the decision process to perform self-management actions. With regard to early exacerbation detection specifically, patients explained a need for tools to substantiate their current health status (Q3). Furthermore, adequate information regarding self-management actions and social support were considered to be important to reduce patients insecurity regarding self-management actions (Q4&5). Finally, patients indicated a need for elimination of barriers in the health care system to reduce the threshold to contact a HCP since they are not always allowed to communicate with their HCP immediately (Q6).

### Potential benefits and barriers of using Mhealth for exacerbation-related self-management

#### *Benefits*

By asking patients about their perspectives towards using mHealth for exacerbation related self-management, several potential benefits were mentioned. Most importantly, patients thought that mHealth could contribute to awareness of symptom deterioration and explaining possible causes (Q7). In their opinion, mHealth could contribute to demonstrating and underpinning of their current health status, which could increase patients’ self-empowerment towards communication with HCPs and achieve that they feel to be heard (Q8). Furthermore, some patients felt that using mHealth could support them in taking adequate actions by reminding them of these actions and thereby eliminating feelings of insecurity and reducing the threshold to contact an HCP (Q9&10). Some patients thought that recently diagnosed patients would benefit most from an mHealth intervention, since they had realized that they had a lack of information at the early stage of their disease. HCPs perspectives towards potential benefits are largely in line with the patients perspectives.

#### *Barriers*

Potential barriers to use mHealth, according to both patients and HCPs, were patients avoiding confrontation with the disease (Q11), preference for personal contact with an HCP (Q12), difficulties with displaying feelings in an application leading to invalid patient measures and lack of trust in advising characteristics of an mHealth intervention (Q13&14). Additionally, already having adequate self-management skills and lack of enthusiasm for mHealth by HCPs, were mentioned as barriers according to patients (Q15&16). Furthermore, an important potential barrier according to HCPs was a potential limited group of eligible patients (Q17). HCPs explained having doubts regarding eligibility based on disease severity, limited health skills and a lack of patient motivation and enthusiasm for self-management as well as for using mHealth.

The HCPs had various perspectives regarding the influence of mHealth on health care contact. In line with the patients perspective, some HCPs were convinced that mHealth could stimulate prompt health care contact, whereas others thought that advising patients through mHealth could lead to postponing health care contact, as it could result in patients seeking information on self-management actions too long themselves (Q18).

### Willingness to use mHealth for exacerbation-related self-management

Based on the patient and HCP responses, the perceived benefits should outweigh the barriers of using mHealth to be willing to use an mHealth intervention. In general, patients who expressed the most needs towards self-management support and perceived mostly benefits of using mHealth, were most willing to use mHealth. In contrast, patients who expressed that they had enough skills to manage exacerbations themselves were less willing to use mHealth for self-management support, as well as patients who perceive many barriers towards using mHealth or have no interest in using mobile technology at all. Some patients who were not using mobile devices stated that they might be willing to use mHealth when it is helpful in managing their disease and when they would be able to learn how to use mobile devices.

### Preferences regarding content of an mHealth intervention

Both patients and HCPs brought in a diversity in preferences regarding the content of an mHealth intervention to support self-management behavior and regarding intensity of mHealth use. All patients and HCPs perceived that an mHealth intervention should be a multi-component and tailored tool that improves self-management of exacerbations by determining their health status to adequately detect exacerbations, provides decision support to overcome barriers to take prompt actions and stimulates prompt healthcare contact.

Both HCPs and patients were positive towards providing information regarding COPD and exacerbation-related self-management, an action plan for decision support and reminders for various activities (Q19–21). Both HCPs and patients perceived potential benefits regarding self-monitoring of symptoms over time, to create awareness on symptom deterioration and subsequently support exacerbation recognition. However, some patients and HCPs explained doubts about patient willingness to enter information in an application for self-monitoring (Q22). Some patients explained that they tried to avoid confrontation with their disease and were therefore not willing to monitor symptoms over time, whereas others were convinced that they could feel best themselves how they are doing and therefore need no self-monitoring tool. Patients mentioned various preferences regarding potential frequency of entering information and corresponding duration.

Some HCPs and patients perceived potential benefits regarding information exchange between patients and HCPs through mobile devices. However, they explained doubts at the same time (Q23). Both patients and HCPs were positive towards a quick link to HCP contact details to support healthcare contact at the moment of symptom deterioration. Most patients expressed that they prefer to make the decision to communicate with their HCP themselves, instead of automatic communication through a mobile device. When talking about information exchange with HCPs, a more in-depth discussion focusing on positioning of the patients and HCPs role regarding mHealth use was elicited, which is described below.

Most importantly, patients and HCPs considered a mHealth intervention as support to improve self-management and emphasized that it should never replace patients’ own feelings nor undermine their own decisions. An mHealth intervention should be complementary to regular contact with HCPs, as personal contact with a HCP was considered to be very important for patients, and for HCPs as well to be able to make adequate medical decisions. Some HCPs expressed that providing medical advice through mobile devices can be unsafe due to the large heterogeneity in patients and symptoms. These HCPS were convinced that a real life judgement on the patient’s health status is necessary to provide adequate medical advice. Based on all focus groups, acceptance of the disease and engagement with mobile device use were considered to be important preconditions for successful outcomes.

### Facilitators for engagement with mHealth

Both patients and HCPs agreed that targeting and tailoring an mHealth intervention is important to optimize engagement with mHealth, although HCPs had their doubts regarding the feasibility of targeting and tailoring (Q24). Some HCPs were equivocal towards the efforts of tailoring an intervention to the patient versus the potential benefits for the patient, as they explained doubts regarding their ability to manage tailored interventions. HCPs emphasized the importance of attractiveness of an mHealth intervention for engagement, meaning that an intervention should be straightforward, not too complex and should include mainly visual information (Q25). Furthermore, both HCPs and patients expressed that receiving positive confirmation or rewards from a mobile device is important. Patients emphasized that an intervention should evoke positive feelings and lead to positive patient outcomes (Q26). All patients emphasized the importance of making their own choices regarding the use of mHealth and that information or feedback should be presented in an advisory manner (Q27). Moreover, an essential precondition from their perspective is that all their HCPs should be familiar with the intervention. In addition, HCPs were convinced that patients should already have some self-management skills and a positive attitude towards using mHealth to optimize engagement with an mHealth intervention (Q28). Finally, safety of an application should be guaranteed at all times.

### HCPs perspectives towards their role regarding mHealth use

HCPs were asked about their perspectives towards their role in supporting self-management using mHealth. They emphasized the importance of adequate positioning of HCPs and patients responsibilities regarding mHealth use (Q29). Most HCPs were critical towards their own role and stated that a pro-active role is needed to achieve positive patient outcomes. Furthermore, most HCPs believe they have a role in estimating patient capabilities to use mHealth for self-management based on patient’s motivation, technical- and self-management skills and cognitive level.

HCPs were asked about potential benefits and barriers regarding patient responsibility toward symptom monitoring and taking action in comparison to monitoring and management by an HCP. One HCP mentioned that increasing patient responsibilities towards managing their disease is preferable and should be a starting point. An important benefit is that it may lead to perceived control on the disease (Q30). However, one HCP mentioned that HCPs have an essential role in judging which actions are appropriate at what time since patients have not enough skills to do that themselves. On the other side, most HCPs were predominantly reluctant regarding symptom monitoring by a HCP. HCPs expressed that monitoring by HCPs could provide a certain feeling of safety by patients which they cannot guarantee. HCPs explained that they will not be able to monitor their patients continuously and were therefore afraid to miss important signals, which might lead to patients who remain waiting until a HCP seeks contact (Q31). Furthermore, patient measures can be invalid, which might result in inadequate (medical) advice and negatively affect patient safety as well. Another barrier for HCPs to monitor patient symptoms through mHealth is the time investment for HCPs (Q32). Finally, HCPs discussed whether it remains self-management support when a HCP is monitoring a patient at home (Q33). Nevertheless, early detection of an exacerbation might be an important potential benefit of symptom monitoring by a HCP.

From the HCPs perspective, an mHealth intervention should be suitable for a wide range of patients and should support them in developing self-management skills over time. The HCPs role should then focus on monitoring how patient self-management skills are developing over time, by discussing patients self-management behavior during consults and providing feedback on self-management skills, leading to future goal setting.

## Discussion

This study provided insight into perceptions of COPD patients and their HCPs towards using mHealth for self-management of exacerbations. This study shows that patients’ needs regarding self-management of exacerbations vary widely and that patients and HCPs perceive mostly similar benefits and barriers regarding using mHealth for exacerbation-related self-management. Patient willingness to use mHealth seems to be driven by these perceived benefits and barriers. Both patients and HCPs are generally positive towards a multi-component and tailored mHealth intervention that aims at developing patients’ self-management skills over time by providing adequate information, decision support regarding prompt actions and feedback on self-management behavior. Although mHealth could support patients in developing self-management skills at home, discussing self-management skills with HCPs in person was considered to be essential for further improvement of these skills.

Several findings of this study were in line with other studies. The patient needs regarding exacerbation-related self-management found in this study correspond with barriers for exacerbation-related self-management that were identified in a previous qualitative study of our research group [27]. Furthermore, we found in this study that using mHealth will remind patients of having COPD, which can be barrier to use mHealth for those patients who try to avoid confrontation with their disease. Huygens et al. described the disadvantage of being reminded of having a chronic condition as well in their study on patient perceptions regarding eHealth for self-management purposes [30]. In line with that study, we found that mHealth should complement the care patients receive from their HCPs, that mHealth should not be too complex and that patients should be allowed to make their own choices regarding whether or not they would like to use mHealth [30]. Moreover, our finding that expected benefits of using mHealth contributes to patient willingness to use mHealth is consistent with the study of Huygens et al. as well [30]. The willingness to use mHealth has been described as an important determinant of actual system use according to the Technology Acceptance Model (TAM) [31]. Our finding that an mHealth intervention should be straightforward and not too complex, corresponds with the ‘ease of use’ determinant of the TAM. Finally, the attitude towards using technology is considered to be import for actual system use according to the TAM, which is in line with our finding that a positive attitude of patients was considered to be important for engagement with mHealth.

Recently, the Behavior Change Wheel has become increasingly important in the development of behavior change interventions [32]. The BCW focuses on identifying relevant intervention functions based on what is understood about the behavior and uses the COM-B (capability, opportunity, motivation and behavior) model [32]. Based on the COM-B model, patients should be capable and have the opportunity and motivation to perform a behavior to achieve behavior change [32]. In line with this model, we found that a minimum level of self-management and technological skills and motivation for mHealth was considered to be important for mHealth use and subsequent behavior change. This supports our finding that not all COPD patients will be eligible for mHealth.

Our findings expand upon prior work by identifying benefits and barriers of using mHealth for exacerbation-related self-management specifically. Our study shows that both patients and HCPs perceived that mHealth could contribute to awareness of symptom deterioration and could help patients in underpinning their current health status, which might increase their self-empowerment and subsequently stimulate prompt health care contact. These are important benefits since previous research has shown that many patients have difficulties with exacerbation detection and taking prompt actions [27, 33, 34].

Although multiple benefits of mHealth were expressed by our participants, this study also shows that both patients and HCPs have doubts regarding the validity of patient measures and have limited trust in advising characteristics of an mHealth intervention. Furthermore, while previous research on action plans aiming at stimulating prompt health care contact, have shown positive outcomes [11, 12], some participants in our study suggested that there might be a chance that an mHealth intervention results in the opposite. By using mHealth a patient might feel more responsible to find out themselves which actions would be adequate to undertake, which could result in postponing health care contact as well. Nonetheless, most participants were convinced that an mHealth intervention can be an important stimulus for prompt health care contact.

Moreover, our study provided insight into patient and HCP preferences regarding content of an mHealth intervention. Based on a previous systematic review, a positive attitude regarding symptom monitoring by a HCP could be expected [35]. However, our study found that both patients and their HCPs have doubts regarding information exchange between patients and HCPs through mobile devices, based on patient and HCP responsibilities towards mHealth use.

### Strengths and limitations

An important strength of this study was our focus on both the patient and healthcare provider perspectives, since both are considered to be very important for intervention development and successful implementation [24]. Furthermore, our study focused on future mHealth opportunities for self-management support which has stimulated participants to reflect on the opportunities of mHealth for self-management in an open manner and express their own ideas, needs and preferences, which provided important insights for future intervention development. Moreover, the trustworthiness of this study was enhanced by using different techniques [29]. Independent moderators were involved in the focus group interviews to guarantee unbiased interviewing of participants and researcher triangulation during data analysis enhanced both the credibility and conformability of the interpretation of the data.

A limitation of this study was the transferability of the results. The panel of HCPs consisted of 6 HCPs with different professions yet lacking an experienced pulmonologist. Most HCP’s had no experience with mHealth in daily practice. Including a larger sample of HCPs with more mHealth experience, could have resulted in a more diverse range of HCP perspectives. However, striving for maximum variation in both the HCP and patient panel contributed to the transferability of the results to other HCP’s and COPD patients. Furthermore, it should be noted that the average age of the study population of COPD patients might be at the lower limit of the Dutch COPD population [36]. Since mobile technology use was most common in the lowest age categories and technological skills were considered to be important for mHealth use, it might be argued whether perceptions regarding acceptability of technology could have been different when including more older patients. The results of this study are relevant for other countries, although the specific health care context and socio-economic level in other countries might influence perspectives regarding mHealth use.

### Implications for practice and future research

The findings of this study are important for both health care providers supporting patients in exacerbation-related self-management as well as researchers focusing on the development of mHealth interventions. The knowledge on barriers towards using mHealth for exacerbation-related self-management might help health care providers to anticipate on potential barriers when using mHealth and to understand related patient behaviors.

This study strengthens the need for multi-component and tailored self-management interventions in COPD care. Although evidence on effectiveness of current mHealth interventions is limited, recent studies suggest that mHealth interventions aimed at supporting self-management might improve patient outcomes [20, 37]. Whilst not reflected in the scientific literature, the rapidly evolving nature of mHealth technologies and their uptake is bound to influence the accessibility and the way we support self-management support in the future, also in patients with COPD. Our study shows that future mHealth interventions should specifically target at developing patients’ self-management skills over time by providing adequate information, decision support regarding prompt actions and feedback on self-management behavior. Furthermore, we should ensure that interventions are attractive, rewarding, safe and tailored to the patients’ needs and that these interventions are complementary to regular care. It needs to be emphasized that, at least for the coming years, not all COPD patients will be eligible for mHealth especially for those with a more negative attitude towards mHealth and with low digital literacy [20]. Given the current trends in internet access and smartphone use [38], also in older populations, this will undoubtedly improve thereafter.

Since usability of mobile devices might depend on the size of devices, it is important that future mHealth interventions can be delivered on a tablet when experiencing problems with navigating on a smartphone. In general, for future development of mHealth interventions it is important to take into account the identified barriers in this study and to meet patient and HCP preferences regarding the content of an intervention. Therefore, both patients and HCPs should be actively involved during the intervention development stage. As self-management requires behavior change, it is important that an mHealth intervention focuses on effective behavior change techniques selected by a thorough analysis of patient capabilities, opportunities and motivation to use mHealth for self-management. Adequate positioning of HCP and patients responsibilities regarding the use of an mHealth intervention to support self-management is essential before implementing an mHealth intervention into COPD care.

## Conclusions

This study provided insight into perceptions of COPD patients and their HCPs towards using mHealth for self-management of exacerbations. The patients willingness to use mHealth seems to be driven by the perceived benefits and barriers of using mHealth. This study points out that future mHealth interventions should focus on developing self-management skills over time by providing adequate information, decision support and feedback on self-management behavior. To optimize engagement with mHealth, it is important that patients have a positive attitude toward using mHealth and that mHealth interventions are attractive, rewarding, safe and tailored to the patient needs. Although mHealth could support patients in developing self-management skills at home, both patients and health care providers believe that the use of mHealth should be complementary to regular care. Future development of mHealth interventions should focus on selecting effective behavior change techniques and take into account the identified potential barriers toward mHealth use identified by this study. Adequate positioning of the HCP and patients role regarding mHealth use is essential before implementing mHealth interventions into COPD care.

## Additional file

Additional file 1: Topic lists for focus group interviews. (DOCX 24 kb)

## Abbreviations

COPD: Chronic Obstructive Pulmonary Disease
HCP: Health Care Provider
mHealth: Mobile Health
