# Supplementary material for: Perceptions of patients with chronic obstructive pulmonary disease and their health care providers towards using mHealth for self-management of exacerbations: a qualitative study
Source: BMC Health Serv Res. 2018 Oct 4;18:757. doi: 10.1186/s12913-018-3545-4 (PMC6172846; doi:10.1186/s12913-018-3545-4)
Supplement: Supplementary file 1 — Topic lists for focus group interviews. (DOCX 24 kb) [file 12913_2018_3545_MOESM1_ESM.docx]

**ADDITIONAL FILE 1**

**Topic list focus group interview with patients**

| **Topic** | **Specification** |
| --- | --- |
| **Introduction** | Aim of the study |
|  | Introduction of important terminology (exacerbation/mHealth) |
| **Use of mobile devices** | Current use of mobile devices |
|  | Perceived advantages & disadvantages |
| **Patient needs** | Patient needs towards self-management (support) of exacerbations |
| **Perceptions towards mHealth** | Perceptions towards use of mHealth for self-management of exacerbations |
|  | Potential benefits and barriers of mHealth |
| **Content of an mHealth intervention** | Initial ideas on content of mHealth intervention |
|  | Initial ideas on design and functions |
|  | Reflection on specific topics:   - Self-monitoring/entering data by patients - Access to data by health care provider - Feedback on behavior - Contact with health care provider - Education - Decision making - Reminders |
| **Intensity of mHealth use** | Perceptions towards intensity of using mHealth |
|  | Perceptions towards daily symptom monitoring |

**Topic list focus group interview with health care providers**

| **Topic** | **Specification** |
| --- | --- |
| **Introduction** | Aim of the study |
|  | Introduction of important terminology (mHealth) |
| **Experience with mHealth in health care** | Current use of mobile technology in health care |
|  | Perceived advantages & disadvantages |
| **Patient needs** | Patient needs towards self-management (support) of exacerbations |
| **Perceptions towards mHealth** | Perceptions towards use of mHealth for self-management of exacerbations |
|  | Potential benefits and barriers of mHealth |
| **Content of an mHealth intervention** | Initial ideas on content of mHealth intervention |
|  | Initial ideas on design and functions |
|  | Reflection on specific topics:   - Self-monitoring/entering data by patients - Access to data by health care provider - Feedback on behavior - Contact with health care provider - Education - Decision making - Reminders |
| **Role of health care provider** | Perceptions towards health care provider role regarding self-management support with mHealth |
|  | Perceptions towards time investment regarding self-management support with mHealth |
|  | Perceptions towards communication between patients and health care providers through mHealth |
